# Validity and reliability of the Persian version of the psychosocial impact of dental aesthetics questionnaire

**DOI:** 10.1186/s12955-019-1188-8

**Published:** 2019-07-18

**Authors:** Navid Naseri, Tahereh Baherimoghadam, Reza Rasooli, Moein Hamzeh, Fahimeh Merikh

**Affiliations:** 1 Department of Orthodontic, School of Dentistry, Shiraz Branch, Islamic Azad University, Shiraz, Iran; 2 Department of Orthodontic, School of Dentistry, Shiraz Branch, Islamic Azad University, Shiraz, Iran; 3 Department of Orthodontic, School of Dentistry, Shahed University of Medical Sciences, Tehran, Iran; 4 Private Practice, Isfahan, Iran; 5 Private Practice, Shiraz, Iran

**Keywords:** Malocclusion, Oral health related quality of life, Cross cultural adaptation

## Abstract

**Background:**

The psychosocial impact of dental aesthetics questionnaire (PIDAQ) is an efficient tool for assessment of oral health-related quality of life (**OHRQoL**). It evaluates the effect of dental esthetics on the psychosocial status of young adults. This questionnaire has been translated to many languages so far. However, it has not yet been translated to Persian. This study aimed to assess the validity and reliability of the Persian version of PIDAQ for use among the young adults.

**Material and method:**

The questionnaire was translated to Persian, back-translated to English and underwent cultural adaptation and pretesting. It was then filled out by 398 young adults (215 females and 183 males) between 18 to 30 years in Shiraz, Iran. The Persian version of PIDAQ along with the index of orthodontic treatment need-aesthetic component (IOTN-AC) and the perception of occlusion (POS) index were administered among participants to assess its discriminant validity.

**Results:**

Factor analysis extracted four domains and the factor loading of domains ranged from 0.479 to 0.837. The Cronbach’s alpha for the Persian version of PIDAQ ranged from 0.809 to 0.886. The mean score for each of the domains and the total score for PIDAQ, classified according to IOTN-AC and POS, showed a significant difference. The mean score acquired by subjects requiring orthodontic treatment was significantly higher than the score acquired by those not requiring orthodontic treatment (*P* = 0.00).

**Conclusion:**

The Persian version of PIDAQ has optimal validity, reliability and responsiveness for assessment of the psychosocial impact of malocclusion on the Iranian young adults.

## Introduction

Quality of life (**QoL**) is a comprehensive concept that includes physical, mental and social aspects of general health of individuals [1, 2]. According to the definition provided by the World Health Organization, **QoL** is defined as one’s perception of their situation in life in terms of culture, value system, goals, expectations, standards and priorities [3]. Living conditions, concerns, expectations, socioeconomic status, health status and political view can all affect the **QoL** of individuals. Thus, in order to assess the **QoL**, influential factors in this respect should be evaluated [4]. **QoL** is a subjective concept not visible to others and is based on individuals’ perception of their different aspects of life; Nonetheless, many authors believe that **QoL** domains should be measurable both subjectively and objectively [5, 6].

Several studies have pointed to the negative effect of impaired dental esthetics on daily life [7, 8]. Oral Health-Rlated Quality of Life **(OHRQoL)** aims to evaluate the etiology of oral diseases, interventions to prevent oral conditions, distribution of oral diseases in different populations, the treatment need and effect of oral conditions on daily activities. This index can also help in allocation of oral health care services [7, 8].

Assessment of the orthodontic treatment need is often based on the occlusion status and cephalometric measurements [9]. The commonly used indices for assessment of occlusion such as the dental esthetic index and index of orthodontic treatment need (IOTN) only evaluate the anatomical structures and malocclusion but do not provide any information about the effect of malocclusion on one’s perception of themselves, **OHRQoL** and daily activities [10]. Thus, they only reflect a professional viewpoint rather than patient expectations. Differences between the attitudes of orthodontists and patients regarding the perception of beauty and dentofacial esthetics as well as the orthodontic treatment need have always caused challenges. The recent interest in **OHRQoL** reveals that a combination of **OHRQoL** assessment tools and occlusal indices are effective for prediction of patients’ orthodontic treatment need [11].

The multiple-item questionnaires are among the most commonly used tools for assessment of **OHRQoL**. The questionnaires used for assessment of **OHRQoL** are divided into two main categories of general questionnaires and generic questionnaires. Generic questionnaires can be used for assessment of **OHRQoL** in many disease conditions and also for assessment of the value of the tool for efficacy analyses. However, these questionnaires have low sensitivity for detection of small differences [12]. The condition-specific questionnaires were designed aiming to further concentrate on specific conditions and populations; These questionnaires are capable of detecting small, but clinically significant, changes [13, 14].

Psychosocial Impact of Dental Aesthetics Questionnaire (PIDAQ) is a multiple-item questionnaire developed in German language, and then published in English language; It was designed as a self-assessment tool for evaluating the effect of dental esthetics on psychosocial status of young adults [15]. This questionnaire can discriminate between different degrees of dental esthetics determined by the IOTN-aesthetic component (IOTN-AC) and the perception of occlusion (POS) index [15].

Before applying questionnaires in different culture and countries, the fulfilment of a translation and validation process that takes the cultural and social aspect of new region into account is essential [16–19]. PIDAQ has been translated to several languages so far and its validity and reliability have been previously confirmed [20–28]. However, this widespread questionnaire has not been translated to Persian yet. This study aimed to assess the validity and reliability of the Persian version of PIDAQ and its cultural adaptation.

## Materials and methods

### Specificity of PIDAQ

PIDAQ is a specific tool for assessment of the psychosocial impact of dental esthetics on young adults. The original version of this questionnaire has 23 questions categorized in four domains including dental self-confidence (6 positive items), social impact (8 negative items), psychological impact (6 negative items) and **aesthetic concern** (3 negative items). A 0–4 five-point Likert scale was used for scoring of each item, scores 0, 1, 2, 3 and 4 indicated “not at all”, “slightly/mild”, “moderate”, “severe” and “very severe”, respectively. Since some of the PIDAQ questions were negative and some others were positive, the scores for dental self-confidence domains including items 1 to 6 were reversed to facilitate the interpretation of results.

Total scores of the negative domains including social impact, psychological impact, **aesthetic concern** and the reversed scores of the positive domain including dental self-confidence were summed to provide the total PIDAQ score. Total PIDAQ score indicate the impact of dental esthetic on psychosocial well-being of patients. Lower scores indicate low effect of dental esthetics on **OHRQoL** while higher score indicate high effect of dental esthetics on **OHRQoL**.

### Translation of PIDAQ

(A) Primary translation: In the first step, three dental students who were native Persian speakers fluent in English separately and independently translated **the published English version** of the questionnaire to Persian. After discussion, the three versions were combined to obtain one Persian version of the questionnaire.
(B) Back-translation: In the second step, two individuals fluent in both Persian and English who held a PhD degree in English literature and were acquainted with the concept of **OHRQoL** back-translated the Persian version of the questionnaire to English and then compared it to the **published English version**. The shortcomings and errors were all corrected, and then the first Persian draft of PIDAQ was prepared as such.
(C) Cross-cultural adaptation: Four experts including two orthodontists, one oral health and community dentistry specialist and a Persian grammarian and linguist thoroughly evaluated the first Persian version of PIDAQ in terms of accuracy, simplicity of the text, grammar, use of proper terms and syntax and expressed their opinion in this respect. Four steps of equivalence namely semantic, idiomatic, experiential and conceptual equivalence have been done for cross cultural adaptation [16].
  Translation of questions #21, 22, and 23 (I don’t like to see my teeth in the mirror, I don’t like to see my teeth in photographs, I don’t like to see my teeth while I look at a video of myself) were meaningless and needed more changes. In Persian language, graded response to a sentences with negative verb is incomprehensible, therefore we replaced the negative verb of these sentences with positive verb which had negative meaning. In this way, negative meaning of sentences was preserved and the sentences became meaningful in Persian language. The second Persian draft of the questionnaire was prepared as such.
(D) Pretest: Prior to the main study, the Persian version of PIDAQ was filled out by 30 medical students between 18 to 30 years to find its shortcomings and improve the clarity of the questions. One of the research team members was present when students filled out the questionnaire to explain any possible ambiguity and record them. The shortcomings regarding the clarity of the questions were corrected and then the questionnaire was administered again among another 30 medical students. After ensuring the accuracy and clarity of the questionnaire, the final Persian version of PIDAQ was prepared (Table 1).

**Table 1:**
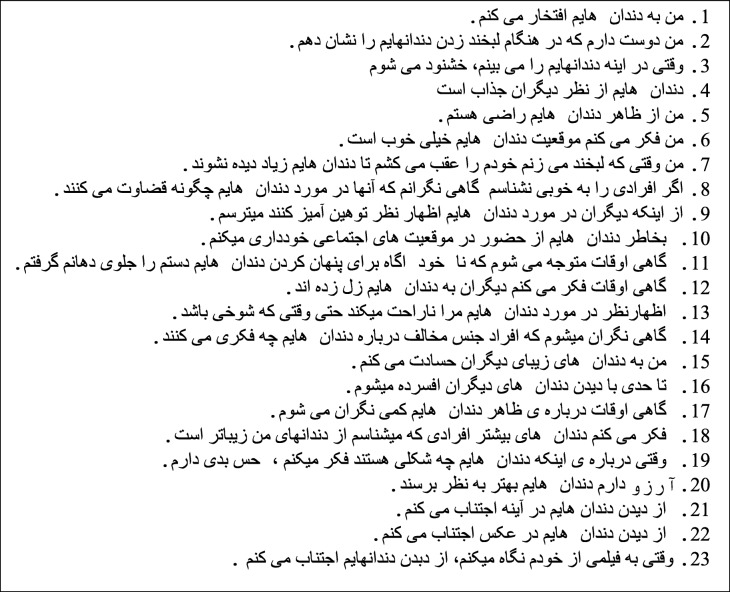
The Persian version of PIDAQ

To assess the validity of the Persian version of PIDAQ and the severity of malocclusion, the IOTN-AC and POS index were used [15].

**IOTN-AC**: This index is used to assess dental esthetics and includes 10 photographs that show different degrees of malocclusion. The participants were instructed to select the photograph which is most similar to their own condition in terms of appearance with no time limitation set.

**POS**: This index has 6 questions regarding dental arrangements and occlusion with special emphasis on dental esthetics. The six items of this index are as follows: There are gaps between the upper front teeth, the upper front teeth are crowded, the lower front teeth are crowded, the upper front teeth are irregular, the lower front teeth are irregular and the upper front teeth are positioned too far anterior to the lower front teeth (the overjet is too large). The scoring of this index, similar to PIDAQ, is based on a 0–4 five-point Likert scale. Scores 0, 1, 2, 3 and 4 indicate “not at all”, “mild”, “moderate”, “severe” and “very severe”, respectively. The sum of scores was then calculated. The total scores of 0–1 indicate personal satisfaction with dental status, scores 2–4 indicate moderate satisfaction, scores 5–8 indicate dissatisfaction and scores > 9 indicate severe dissatisfaction of subjects with their dental status.

### Need for orthodontic treatment

The participants were asked if they needed orthodontic treatment to correct their teeth with response options “Yes” or “No”.

In order to assess the validity and reliability of the Persian version of PIDAQ, medical student between 18 to 30 years residing in Shiraz, Iran were asked to participate in the study. The exclusion criteria were as follows:

- Those with mental, physical or psychological disorders
- Those with Craniofacial anomaly
- Those with carious, missed or fractured teeth in the anterior region
- Those with discolored teeth, moderate to severe fluorosis or dental staining in the anterior region
- Subjects with a previous history of orthodontic treatment
- Subjects with a history of esthetic dental treatments (i.e. laminate veneers, etc.) in the anterior region

### Statistical analysis

SPSS version 18.0 (SPSS Inc., Chicago, IL, USA) was used for data analysis. Normal distribution of variables was evaluated using the Kolmogorov-Smirnov test. The sample size was calculated by using Bonnett’s Formula. Factor analysis was applied using principle components analysis and Varimax rotation to assess the construct validity. Kaiser-Meyer-Olkin Measure of sampling adequacy was performed before factor analysis to determine the capacity of the variables for inclusion in factor analysis. The internal consistency of the Persian version of PIDAQ was tested using the Cronbach’s alpha coefficient for the subscales.

The discriminant validity was tested by comparing the total score of PIDAQ and scores of its domains with self-reported IOTN-AC and POS via one-way ANOVA (Analysis of variance) and the Kruskal-Wallis test. Difference of scores between two groups (“have demand” or “no demand” for orthodontic treatment) was assessed by using independent samples t-test.

Test-retest reliability was evaluated with two-way random effects model for 30 randomly selected subjects who filled out the questionnaire for the second time after a two-week interval.

Floor and ceiling effect within each subscale was determined by the percentage of the achieved lowest and highest numeric value of a score. Floor and ceiling effects are considered present when more than 15% of the individuals achieve these values. Presence of floor and ceiling effects is indicative extreme items are missing in the lower or upper end of the scale and limited content validity.

## Results

A total of 400 individuals participated in this study; out of which, 398 (mean age, of 26 ± 0.56 years) returned questionnaires with no missing data. Two of participants just filled out demographic questions. Therefore, they were excluded from study. Sex distribution was 54% (*n* = 215) female and 46% (*n* = 183) male. Participants were between 18 to 30 years (mean age of 26 ± 0.56 years). Of all participants, 27.5% expressed that they required orthodontic treatment.

### Construct validity

The Kaiser-Meyer-Olkin Measure of Sampling adequacy was calculated to be 0.931 and the Bartlett’s test result was significant (*p* < 0.001). These results revealed that the variables were normally distributed and suitable for inclusion in factor analysis. In factor analysis, four factor domains were extracted with the item factor loading ranging from 0.479 to 0.837 (Table 2).

**Table 2:** Factor loadings of the PIDAQ item scale scores after principal component analysis and orthogonal rotation

	Dental self-confidence	Social impact	Psychological impact	Aesthetic concern	Cronbach’s Alpha if Item Deleted
1. I am proud of my teeth.	0.784^*^	0.007	0.174	0.162	0.861
2. I like to show my teeth when I smile.	0.655^*^	−0.036	−0.076	0.119	0.897
3. I am pleased when I see my teeth in the mirror.	0.837^*^	0.051	0.085	0.183	0.851
4. My teeth are attractive to others.	0.792^*^	0.092	0.159	0.135	0.861
5. I am satisfied with the appearance of my teeth.	0.801^*^	0.193	0.285	0.100	0.851
6. I find my tooth position to be very nice.	0.733^*^	0.185	0.226	0.050	0.870
7. I hold myself back when I smile so my teeth don’t show so much.	0.300	0.581^*^	0.058	0.381	0.826
8. If I don’t know people well, I am sometimes concerned about what they might think about my teeth.	0.162	0.658^*^	0.184	0.349	0.829
9. I am afraid that other people could make offensive remarks about my teeth.	0.110	0.724^*^	0.283	0.032	0.880
10. I am somewhat inhibited in social situations because of my teeth.	0.051	0.646^*^	0.090	0.401	0.840
11. I sometimes catch myself holding my hand in front of my mouth to hide my teeth.	0.135	0.492^*^	0.158	0.469	0.832
12. Sometimes, I think people are staring at my teeth.	−0.186	0.479^*^	0.169	0.327	0.854
13. Remarks about my teeth irritate me even when they are meant jokingly.	0.110	0.602^*^	0.503	−0.071	0.828
14. I sometimes worry about what members of the opposite sex think about my teeth.	0.081	0.594^*^	0.425	0.264	0.835
15**.** I envy the nice teeth of other people.	0.071	0.061	0.737^*^	0.199	0.818
16. I am somewhat distressed when I see other people’s teeth.	0.067	0.283	0.626^*^	0.317	0.874
17. Sometimes I am somewhat unhappy about the appearance of my teeth.	0.249	0.408	0.551^*^	0.234	0.871
18. I think that most people I know have nicer teeth than I do.	0.271	0.230	0.576^*^	0.317	0.881
19. I feel bad when I think about what my teeth look like.	0.269	0.391	0.575^*^	0.369	0.874
20. I wish my teeth looked better.	0.193	0.328	0.664^*^	0.050	0.870
21. I don’t like to see my teeth in the mirror.	0.170	0.313	0.299	0.583^*^	0.874
22. I don’t like to see my teeth in photographs.	0.267	0.037	0.341	0.684^*^	0.865
23. I don’t like to see my teeth while I look at a video of myself.	0.193	0.217	0.219	0.766^*^	0.874
Variance Explained (Initial solution)	9.036	2.824	1.257	0.937	
% of Variance Explained (Initial solution)	39.287	12.280	5.465	4.073	
% of Variance Explained (rotation solution)	18.017	15.303	14.821	12.964	
Cumulative % of Variance Explained (rotation solution)	18.017	33.320	48.141	61.105	

*P* < 0.05 *

First extracted domain contained items 1–6, comprising dental self-confidence subscale and explained 39.29% of the variance. Second extracted domain contained social impact subscale items 7–14 and explained 12.28% of the variance. Third extracted domain contained 15–20 items as the Psychological Impact subscale and explained 5.47% of the variance. The last extracted domain contained the items 21–23 of **the aesthetic concern** subscale and explained 4.07% of the variance. These 4 domains together explained 61.11% of the total variance (Table 2).

### Reliability

The Cronbach’s alpha coefficient of internal consistency was calculated to be 0.925. The standard Cronbach’s alpha coefficient was 0.927. The Cronbach’s alpha coefficient was 0.809 for the **aesthetic concern**, 0.830 for social impact, 0.853 for Psychological impact, and 0.886 for the dental self-confidence. The test-retest correlation coefficient was 0.982, 0.782, 0.812, and 0.716 for dental self-confidence, social impact, psychological impact, and **aesthetic concern**, respectively.

### Discriminant validity

Mean scores of PIDAQ subscales and total score increased gradually with increasing the severity of malocclusion. There was a statistically significant difference in dental self-confidence, social impact, Psychological Impact, aesthetic concern subscales and total scores as categorized by IOTN-AC and POS (Tables 3 and 4), as well as the reported orthodontic treatment demand (Table 5).

**Table 3:** Subscales and PIDAQ scores according to POS categorization

POS Scores	Dental self-confidence (reverse scores of items 1–6)	Social impact (score of items 7–14)	Psychological impact (score of items 15–20)	Aesthetic Concern (scores of items 20–23)	Total
0–1(*n* = 171)	8.13 ± 5.29	6.21 ± 5.31	6.53 ± 4.61	2.15 ± 2.22	23.02 ± 12.50
2–4(*n* = 124)	10.42 ± 4.82	8.90 ± 5.41	8.42 ± 5.25	4.12 ± 2.15	29.87 ± 13.10
5–8(*n* = 59)	11.23 ± 4.50	10.10 ± 6.32	9.54 ± 3.41	6.42 ± 3.91	37.29 ± 17.21
9 < (*n* = 46)	13.17 ± 4.65	13.42 ± 6.70	10.60 ± 4.10	8.61 ± 5.21	45.80 ± 15.85
ANOVA	0.00 **	0.00 **	0.00 **	0.00 **	0.00 **
Spearman correlation	*r* = −0.378 0.00 **	*r* = −0.215 0.00 **	*r* = −0.2260.00 **	*r* = − 0.375 0.00 **	*r* = − 0.295 0.00 **

** *P* < 0.01

**Table 4:** Subscales and PIDAQ scores according to IOTN-AC categorization

IOTN-AC Score	Dental self-confidence (reverse scores of items 1–6)	Social impact (score of items 7–14)	Psychological impact (score of items 15–20)	Aesthetic Concern (scores of items 20–23)	Total
1 (*n* = 222)	6.13 ± 5.12	7.24 ± 5.22	5.51 ± 5.42	2. 81 ± 2.12	21.69 ± 15.62
2 (*n* = 88)	7.14 ± 5.12	8.56 ± 4.21	6.69 ± 3.74	3.55 ± 3.13	25.96 ± 13.39
3 (*n* = 43)	11.32 ± 6.12	10.22 ± 3.49	8.11 ± 5.19	7.24 ± 5.91	36.89 ± 15.21
≤ 4 (*n* = 47)	13.21 ± 5.42	11.45 ± 3.42	12.15 ± 6.12	8.31 ± 5.18	45.13 ± 16.72
ANOVA	0.00 **	0.00 **	0.00 **	0.00 **	0.00 **
Spearman correlation	r = −-0.312 0.00 **	r = − 0.352 0.00 **	r = −0.252 0.00 **	r = −-0.282 0.00 **	r = − 0.410 0.00 **

** *P* < 0.01

**Table 5:** Comparison of the PIDAQ item scale scores according to orthodontic treatment need

	Demand for orthodontic treatment			Do not Demand for orthodontic treatment			
	Total mean score			Total mean score			*P*-value
	Female	Male	*P*-value	Female	Male	*P*-value	
Dental self-confidence	12.01 ± 5.21			8.54 ± 5.12			0.00**
	11.57 ± 3.40	12.03 ± 4.01	0.412	9.15 ± 5.53	7.99 ± 4.98	0.021*	
Social impact	14.11 ± 4.15				12.57 ± 3.76		0.00 **
	14.12 ± 4.91	15.03 ± 3.85	0.451	12.54 ± 4.70	10.03 ± 6.41	0.11*	
Psychological impact	12.43 ± 4.18			9.82 ± 5.91			0.00 **
	12.91 ± 4.40	12.11 ± 4.74	0.812	10.21 ± 4.65	9.61 ± 5.77	0.737	
Aesthetic Concern	7.21 ± 3.52			5.01 ± 4.32			0.00 **
	6.74 ± 3.41	7.47 ± 3.81	0.321	5.64 ± 3.55	4.86 ± 4.17	0.892	

*P < 0.05, ** *P* < 0.01

No Floor and ceiling effects were found in this study. Ceiling or floor effects were considered not to be present as the percentages did not exceed 15%.

## Discussion

Several factors can affect the growth and development of the jaws and lead to malocclusion including genetics, congenital anomalies, systemic diseases and environmental factors [29]. Studies on the psychological impact of malocclusion on the OHRQoL are increasing, which highlights the significant role of an esthetic appearance in social relations [30]. However, the conception of dental clinicians and patients of dental esthetics does not always match. Tools such as questionnaires can be employed by dental clinicians to better understand the patients’ cognitive needs and expectations with respect to their dental esthetics.

The PIDAQ is a multi-item self-reported tool which was designed to assess dental esthetics on the psychosocial status of young adults; It was developed in German language and then published in English language [15]. In order to use PIDAQ in different language and cultural context, translation and revalidation is essential.

Translation of PIDAQ to Persian included four steps of accurate translation, back-translation, cross-cultural adaptation and pretesting. Cross-cultural adaptation is a process to assess the equivalence of the English and translated versions of a questionnaire. To assess the equivalence of a translated questionnaire to its English version, four steps of equivalence should be considered namely semantic, idiomatic, experiential and conceptual equivalence [16]. Semantic equivalence assess equivalence in the meaning of words and determines the present problem with grammar and vocabulary. Idiomatic equivalence is concerned about colloquialisms or idioms which are difficult to translate. Experiential equivalence evaluates whether the situations evoked or depicted in the original version are fit the target cultural context. Conceptual equivalence is concerned the words which hold different conceptual meaning between cultures. In this study, a committee composed of two orthodontists, one oral health and community dentistry specialist and one Persian language linguist and grammarian examined the translated and back-translated questionnaires for these four types of equivalence items. They evaluated the Persian version of PIDAQ in terms of simplicity of the text, language grammar, use of proper terms and syntax.

Translation of questions #21, 22 and 23 (I don’t like to see my teeth in the mirror, I don’t like to see my teeth in photographs, I don’t like to see my teeth while I look at a video of myself) required more changes in order to be understandable in Persian language. The graded responses to sentences with a negative verb (I don’t like to see …) in the Persian language is incomprehensible and could mislead the participants. Therefore, we had to use positive verb with negative meaning in theses sentences. After first pretest, the opinions and suggestions of the participants were also greatly used to improve the clarity and understandability of the questions and eliminate the ambiguities. Pretesting was repeated, which showed that the translated questions were acceptable.

Assessment of the internal consistency of the Persian version of PIDAQ indicated optimal internal reliability. The Cronbach’s alpha was calculated to be 0.925. The lowest value belonged to the questions regarding “**aesthetic concern**” while the highest value belonged to “psychological impact”. The Cronbach’s alpha reported in Chinese and Nepalese populations was also high, similar to our study, while it was lower in Spanish, Brazilian and Turkish populations [20–24]. The Cronbach’s alpha in our study was slightly higher than the value reported for the **published English version** of PIDAQ (0.86 to 0.91), which indicates similar results for the Persian version and original version of the questionnaire [15].

The Cronbach’s alpha in our study did not significantly change following elimination of any of the questions, which highlights the fact that all questions had optimal internal consistency. The retest reliability for each of the domains of self-confidence, social impact, psychological impact and **aesthetic concern** was found to be 0.982, 0.782, 0.812 and 0.716, respectively, which indicated optimal retest reliability of the Persian version of the questionnaire.

In the present study, factor analysis was performed to assess the construct validity. Factor analysis extracted three domains from 23 questions. The first domain included the same questions of the dental self-confidence domain of the original version of PIDAQ. The second domain, which was first referred to as the aesthetic concern, included all items regarding the psychological impact and **aesthetic concern** in the **published English version** of the questionnaire. The items in the third domain included all questions of the social impact domain of the original version. Since the factor analysis in the original study extracted four domains, we tried our best to also extract four domains by factor analysis. After adjusting the factor analysis on four domains, the first domain included questions 1 to 6, the second domain included questions 7 to 14, the third domain included questions 15 to 20 and the fourth domain included questions 21 to 23. The first domain, which was referred to as dental self-confidence as in the original version, indicated the effect of dental esthetics on subjective feelings and perception of individuals of their well-being and showed the highest rate of explained variance among other extracted domains.

The cumulative contribution of the four domains extracted from the Persian version of PIDAQ was less than the cumulative contribution of the **published English version** of the questionnaire. This indicates the loss of information by slightly higher than one-third. Different cultural background can, to some extent, explain the slight difference between the results of factor analysis and the extracted domains from the Persian version compared to the published English version. However, extraction of four domains from the Persian version of PIDAQ can show information in four domains. Thus, the questionnaire had good construct validity and completely satisfactory content validity.

The discriminant validity of the Persian version of PIDAQ questionnaire was evaluated by assessing the correlation between the four extracted domains from the Persian version and using IOTN-AC and POS indexes. A significant correlation was noted between different scores of PIDAQ and its subscales with the used indices. However, IOTN-AC has been extensively used in the Iranian population [31]. Despite the afore-mentioned limitation of IOTN, it has also been used in studies that translated PIDAQ to Turkish and Chinese languages and also in the study introducing the **published English version** of PIDAQ [15, 20, 22]. The current results showed that the Persian version of PIDAQ could discriminate between different scores of IOTN and POS, similar to the **published English version** of the questionnaire.

In the present study, all participants were divided into two groups based on their orthodontic treatment need according to their self-expressed opinion. The total score of PIDAQ and the four extracted domains were totally different between the two groups with and without the demand for orthodontic treatment, which indicates the optimal responsiveness of PIDAQ in males and females. Nonetheless, it was found that females not requiring orthodontic treatment acquired a significantly higher score in dental self-confidence and social domain compared to males, which may be due to the greater sensitivity of females to aesthetic issues. This finding was in agreement with the results of a study on the Turkish population [22].

## Conclusion

The Persian version of PIDAQ showed optimal validity and reliability when tested on subjects in Shiraz city. It is a responsive tool for assessment of the psychological impact of dental esthetics in a community. This questionnaire is highly reliable and valuable for researchers focusing on the orthodontic-related QoL.

## Data Availability

The datasets used and/or analysed during the present study are available from the corresponding author.

## Abbreviations

ANOVA: Analysis of variance
IOTN: Index of orthodontic treatment need
IOTN-AC: Aesthetic component of the index of orthodontic treatment need
OHRQoL: Oral health related quality of life
POS: Perception of occlusion scale
QoL: Quality of Life
